# Parameter efficient multi-model vision assistant for polymer solvation behaviour inference

**DOI:** 10.1038/s41524-025-01658-7

**Published:** 2025-05-31

**Authors:** Zheng Jie Liew, Ziad Elkhaiary, Alexei A. Lapkin

**Affiliations:** 1 Department of Chemical Engineering and Biotechnology, University of Cambridge, Cambridge, CB3 0AS UK; 2 Yusuf Hamied Department of Chemistry, Innovation Centre in Digital Molecular Technologies, University of Cambridge, Cambridge, CB2 1EW UK

**Keywords:** Materials science, Soft materials, Polymers

## Abstract

Polymer–solvent systems exhibit complex solvation behaviours encompassing a diverse range of phenomena, including swelling, gelation, and dispersion. Accurate interpretation is often hindered by subjectivity, particularly in manual rapid screening assessments. While computer vision models hold significant promise to replace the reliance on human evaluation for inference, their adoption is limited by the lack of domain-specific datasets tailored, in our case, to polymer–solvent systems. To bridge this gap, we conducted extensive screenings of polymers with diverse physical and chemical properties across various solvents, capturing solvation characteristics through images, videos, and image–text captions. This dataset informed the development of a multi-model vision assistant, integrating computer vision and vision-language approaches to autonomously detect, infer, and contextualise polymer–solvent interactions. The system combines a 2D-CNN module for static solvation state classification, a hybrid 2D/3D-CNN module to capture temporal dynamics, and a BLIP-2-based contextualisation module to generate descriptive captions for solvation behaviours, including vial orientation, solvent discolouration, and polymer interaction states. Computationally efficient, this vision assistant provides an accurate, objective, and scalable solution in interpreting solvation behaviours, fit for autonomous platforms and high-throughput workflows in material discovery and analysis.

## Introduction

Polymer solvation behaviours^1^ such as complete dissolution^2^, swelling^3^, and gelation^4,5^, along with colloidal dispersions^6^, are critical across various industries due to their influence on material applications and performance. Unlike simpler dissolution processes in non-polymeric materials, polymer–solvent interactions are inherently complex, involving solvent diffusion into the polymer matrix^7^, polymer chain disentanglement^8^, and structural changes. While predictive methods such as the Flory–Huggins theory^9^ and Hansen solubility parameters^10^ offer insights into general solubility and miscibility, they often fall short in capturing the dynamic and nuanced solvation behaviours^11^, including partial interactions and morphological transitions. Manual visual screening of polymer–solvent systems remains widely used due to its practicality.

Assessing polymer–solvent interactions has traditionally relied on a range of methods^12^, each with inherent limitations^13^. Visual inspection of cloud points, a widely used qualitative approach, is inherently subjective and prone to observer bias^14,15^. Quantitative methods, such as intrinsic viscosity measurements commonly used in polymer recycling, require controlled experimental conditions, limiting their applicability to simple systems^16^. Despite the existence of quantitative methods, many laboratories still depend on visual assessment in inferring solvation states, observing changes, such as transparency, colour shifts, or gel formation. Such observation relies on human interpretation, which introduces subjectivity and inconsistencies. As a result, methods based on human inference are less suitable for high-throughput screening workflows^17^ and not suitable for automated platforms^18,19^, where accurate and efficient analysis is essential.

Recent advancements in computer vision have introduced new opportunities for vision-enabled analysis for studying material sciences^20^. Convolutional neural networks (CNNs)^21,22^ have revolutionised machine vision, enabling applications such as recognising laboratory apparatus for accurate classification^23^ and solubility assessments to determine whether a solute–solvent state is dissolved or undissolved^24^. Furthermore, computer vision methods have been employed to interpret turbidity, solid formation, and liquid levels, providing real-time feedback for chemical reactions^25^. However, 2D-CNNs have primarily been employed for generic classification tasks and have yet to be applied for discerning subtle, static variations in material conditions, such as polymer solvation states, where nuanced morphological and compositional differences require more domain-specific approaches.

The necessity of a video-based input approach arises when analysing dynamic phenomena in material behaviour, including phase changes, particular dispersion, colloidal systems, and viscous transformations. Extending beyond image-based capabilities, 3D-CNNs^26^, originally designed for action recognition, hold significant potential for applications in dynamic material analysis. By leveraging spatial–temporal filters^27^, 3D-CNNs encode temporal dynamics, enabling the interpretation of transitions in physical states or chemical processes over time. Despite this potential, their use has largely been confined to generic action and activity recognition tasks, such as human action monitoring^28^ and laboratory activity detection^29^, with limited exploration in domain-specific applications like material science.

The established method of training CNNs involves leveraging pretrained models^30,31^ fine-tuned on an application-specific dataset. This strategy is widely adopted to minimise training time and computational demands by transferring knowledge from large-scale datasets. Unlike 2D-CNNs, the computationally intensive nature of 3D-CNNs poses significant challenges for feasible implementation^32^, as they possess prolonged inference times due to their spatial–temporal processing, making them impractical for deployment in self-driving laboratories and high-throughput screening workflows. In such environments, where human-in-the-loop processes^33^ rely on rapid and efficient analysis, these limitations underscore the need for more parameter-efficient and inference-friendly models.

Neither 2D-CNNs nor 3D-CNNs have yet been applied specifically to discern polymer solvation behaviours. This gap is likely due to the absence of domain-specific and visually diverse datasets tailored to capturing the nuanced behaviours of polymers in solvents. Polymer solvation encompasses a variety of complex phenomena, including dissolution, gel formation, swelling, and dispersion, all of which require specialised datasets. Without such datasets, it is difficult for CNNs to learn the unique visual cues produced through polymer–solvent interactions. Addressing this limitation by introducing novel methodologies and domain-specific datasets could unlock significant opportunities for using computer vision for polymer solvation analysis.

Beyond classification of polymer solvation states, contextualisation is essential for capturing the intricacies of morphological changes such as degree of dissolution, extent of swelling, and phase separation dynamics. Vision-language models (VLMs)^34,35^ have emerged as a promising approach, enabling the integration of visual data with textual information. Among these, models such as contrastive language-image pretraining (CLIP)^36^ are recognised for their ability to align images with textual labels. However, their capabilities are primarily focused on retrieval tasks^37,38^ and lack the generative semantic depth required for applications like contextualised captioning. In contrast, bootstrapping language-image pre-training (BLIP)^39^ and its advanced iteration, BLIP-2^40^, excel in generating precise, contextually rich descriptions by learning shared visual–textual representations. Despite its potential, applications of BLIP in material analysis remain underexplored, largely due to the lack of domain-specific image–text caption datasets, which has hindered the broader adoption of VLMs for autonomous material behaviour investigations.

Analysing polymer solvation behaviours poses significant resource challenges due to the variety of states that unique polymer–solvent combinations can exhibit. This work presents a parameter-efficient multi-modular vision assistant (VA) designed to facilitate accurate inference and contextualisation of polymer solvation behaviour. The VA integrates computer vision and vision-language capabilities through a programmable camera interface, addressing subjectivity of manual inference while reducing labour requirements. Trained with a domain-specific polymer–solvent interaction dataset generated from a high-throughput screening workflow developed in-house, the VA model enables static and dynamic inference and classification of polymer solvation behaviours, with the ability to contextualise the visual states of both the polymer and solvent. We first leverage a 2D-CNN to build the static inference module, which interprets and classifies polymer solvation conditions through images. Next, a hybrid modelling approach is employed, where feature vectors extracted from the 2D-CNN enable domain-specific training of a 2D-/3D-CNN model for dynamic inference. This hybrid model captures temporal dynamics and complex interactions, identifying simultaneous and evolving phenomena that become apparent with vial movement. Finally, we incorporate contrastive learning by fine-tuning vision-language models to establish image–text correlations. This approach enables contextualisation, allowing the VA to assess additional characteristics beyond polymer solvation behaviour, including identification of vial orientation, solvent colour and intensity, and external observational cues such as phase separation patterns or surface reflections.

## Results

### Vision assistant model description

Polymer-solvation states can be represented in myriad of ways, reflecting the complexity and variability of interactions between polymers and solvents. For the purposes of this study, five distinct polymer solvation states are defined, namely dissolved, undissolved, swelling, gelation, and dispersion states. These classes capture unique aspects of solvation states, reflecting critical phenomena such as molecular solubility, phase transitions, and polymer structural changes, which are essential for understanding and optimising material properties. The VA is built on a domain-specific polymer solvation dataset developed for this study, ensuring that the model’s insights are directly relevant to real-world applications.

**Fig. 1:**
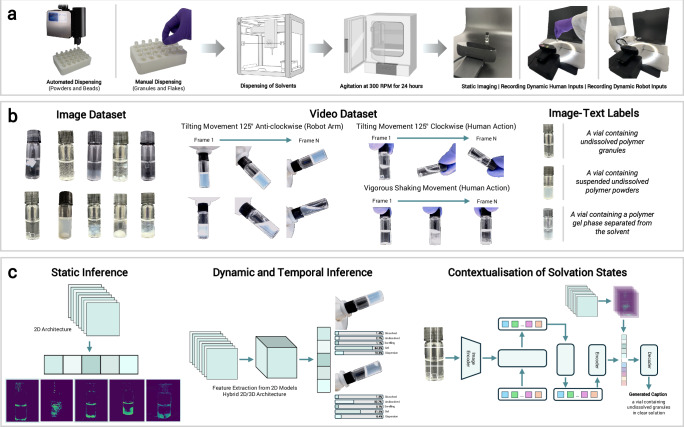
Experimental screening, dataset generation, and multi-modular vision assistant. **a** High-throughput experimental workflow for screening polymer solvation behaviours. Polymers in powder or bead forms are dispensed using a solid dosing machine while flakes and granules are manually distributed into vials. Solvents are dispensed into the vials using a liquid handling machine to achieve a concentration of 2 wt/vol%. Vials are agitated at 300 RPM for 24 h, followed by static imaging to capture polymer solvation states. Handling techniques, including vigorous shaking, manual tilting of vials clockwise (110°), and robotic tilting anticlockwise (110°), are employed to investigate dynamic polymer behaviours. **b** A snippet of polymer solvation dataset, consisting of over 13,663 static images representing distinct solvation states, 5053 dynamic video files capturing dynamic interaction characteristics, and 8011 labelled polymer–solvent image–text pairs for training the contextual vision-language model. **c** The vision assistant comprises three modules: (i) a static inference module using a 2D-CNN for feature extraction and classification of solvation states; (ii) a dynamic inference module employing a novel hybrid 2D feature-extracted 3D-CNN to classify temporal and simultaneous phenomena with high accuracy and efficient training, bypassing the need for publicly available pretrained models; and (iii) a vision-language module trained on pre-labelled image–text pairs to contextualise solvation states and infer frames from video inputs.

The VA consists of three interconnected modules, each designed to interpret unique aspects of polymer solvation behaviour. The static inference module employs a 2D-CNN architecture to discern static solvation states, with fine-tuned pretrained models proving most effective. These models achieve high accuracy, capturing the detailed features from the image dataset generated during the screening workflow (Fig. 1a, b). To support the more parameter-intensive modules, we developed a compute-efficient approach to extract feature maps from the 2D-CNN models, capturing feature vectors for integration into the dynamic inference and contextualisation tasks. The dynamic inference module builds on this approach, using the 2D-CNN model as a feature extractor before incorporating 3D convolutional layers to understand temporal changes in video sequences, forming a hybrid 2D/3D-CNN architecture (Fig. 1c). Developed to capture time-dependent phenomena and solvation state transitions, the dynamic module in the VA can distinguish behaviours such as swelling progression and discriminate dispersive from gel conditions. By combining spatial features extracted from individual frames with temporal modelling across sequences, the hybrid architecture provides enhanced accuracy in identifying complex and evolving behaviours while maintaining computational efficiency. Beyond discrete classification, the contextualisation module incorporates a fine-tuned vision-language model to enhance the interpretability of solvation behaviours by simulating human-like descriptions. It employs a concatenation strategy to combine text embeddings from the VLMs with feature vectors from convolutional maps, improving the alignment of vial images with experimental context. Variations within the same class, such as high turbidity versus slight cloudiness, and changes in characteristics like densities and colour, can be inferred efficiently. While VLMs may have relatively higher computational overhead, their integration is selective and focused on contextualisation rather than classification, complementing the other modules uniquely.

### Static inference

**Fig. 2:**
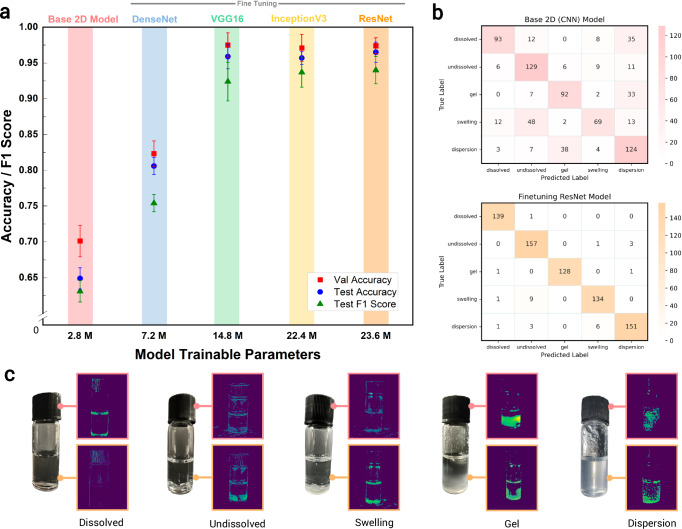
Performance benchmarking and feature map visualisation of 2D-CNN static inference models. **a** Validation and test performance for a five-class classification task of the 2D-CNN baseline model (in figure, named base 2D model) without fine-tuning pretrained models, and pretrained fine-tuned models: DenseNet, VGG16, InceptionV3, and ResNet50. The total number of trainable parameters are used as a metric to compare between the models. **b** Confusion matrices comparing between the best pretrained fine-tuned model, ResNet50 and the 2D-CNN baseline model without fine-tuning. **c** Feature maps generated from the static inference model from the initial interpretable layers of both the base model and the fine-tuned ResNet50 model.

The static inference module employs 2D-CNN architectures^41^ to classify solvation states from static vial images. Figure 2a shows benchmarking results for models of varying complexity, illustrating the trade-offs between computational cost and accuracy. A baseline 2D-CNN model with 2.8 million trainable parameters achieves a validation accuracy of 71.2% and a test accuracy of 65.7%. Fine-tuned pretrained architectures significantly outperform the base model, with DenseNet^42^ (7.2 million parameters) achieving a validation accuracy of 84.1% and a test accuracy of 80.5%. Larger architectures like fine-tuned VGG16^28^, InceptionV3^43^, and ResNet50^44^ (14.8, 22.4, and 23.6 million parameters, respectively) achieve validation and test accuracies exceeding 95%.

While the fine-tuned models demonstrate exceptional accuracy, their computational cost scales linearly with the number of parameters. Figure 2b provides confusion matrices comparing the baseline 2D-CNN model with the fine-tuned ResNet50 model, the latter achieving near-perfect classification. The base model struggles particularly with challenging solvation states, such as distinguishing between gels and dispersions. These two classes often exhibit similar appearances, such as murky or cloudy vials, but differ fundamentally in their mechanical properties: gels resist flow and have solid-like structures, while dispersions consist of freely suspended particulates. The ResNet50 model, by contrast, shows minimal confusion between these states, demonstrating its capability to learn and utilise more distinctive features.

Misclassification of swelling and undissolved states is another challenge, especially for the baseline model, which frequently classifies the former as the latter. Even the ResNet50 model shows occasional misclassification between these classes, although undissolved states are rarely labelled as swollen. This asymmetry indicates that the model is more sensitive to the distinct characteristics of swollen polymers, which often involve subtle changes in morphology or optical properties. This challenge is not unique to machine learning models; even with human interpretation, it is often difficult to reliably distinguish between swelling and undissolved states unless clear signs, such as significant volume expansion or colour changes due to solvent penetration effects, are present. These difficulties highlight the limitations of static inference, as accurate classification often requires additional context, such as the polymer’s initial conditions before solvent addition.

Feature maps from the initial Conv2D layers, shown in Fig. 2c, provide interpretable insights^45,46^ into the learned features of the static inference model, revealing recognisable patterns such as edges, textures, or granular distributions. While the base 2D-CNN model demonstrates higher classification accuracy compared to the fine-tuned ResNet50 model for discrete solvation classes, quantitative accuracy alone does not provide a complete picture. Over-reliance on numerical accuracy can be misleading, as models may overfit or memorise patterns rather than learning generalisable representations. Examining interpretable feature maps from these models offer deeper insights into how they differentiate solvation states that contributes to their decision-making processes. For instance, undissolved polymers are characterised by granular structures, while dispersed states exhibit scattered particulates that create a translucent or cloudy appearance. Static images alone often fail to fully capture the distinctions between complex solvation behaviours, such as gels versus dispersions or undissolved versus swelling states, which require temporal context. This analysis highlights the importance of feature interpretability over model accuracy alone and supports the validity of using 2D-CNN models as feature extractors for the dynamic inference module, enabling the capture of spatiotemporal phenomena and improving system performance. Further, we gain insights into the models’ ability to identify meaningful patterns, which can then be used as feature embeddings for image–text embedding interpretations, linking visual patterns to descriptive solvation states.

### Dynamic, temporal, and simultaneous inference

The dynamic inference module addresses the limitations of static inference by incorporating temporal and behavioural context to interpret polymer solvation states. While 2D-CNN models demonstrate the ability to classify static vial images, they are inherently limited in distinguishing solvation states that require an understanding of changes over time. A vial containing a clear solution might be interpreted as a dissolved condition but could instead contain a transparent polymer gel, distinguishable only by its resistance to flow under rotational or tilting movements. Similarly, a vial containing a solvent with suspended polymer particles might initially appear to be a dispersion; however, over time, those particles could settle at the bottom, indicating an undissolved state.

Traditional 3D-CNN training approaches often rely on pretrained architectures with millions of parameters, learning generic features such as backgrounds and common objects, as seen in models like R3D^47^ or C3D^48^. While effective for general tasks, these models are computationally intensive and less suited for domain-specific applications with constrained datasets and limited computational resources. Hybrid 2D-/3D-CNN models, primarily used in healthcare imaging^49^ and action recognition^50^, have demonstrated efficiency in tackling video summarisation tasks^51,52^. We demonstrate that by using a 2D-CNN model as a feature extractor, we can extend it across the temporal dimension by layering a 3D-CNN on top, resulting in a 2D-/3D-CNN hybrid that significantly reduces computational requirements while maintaining exceptional accuracy in the classification of solvation states.

**Fig. 3:**
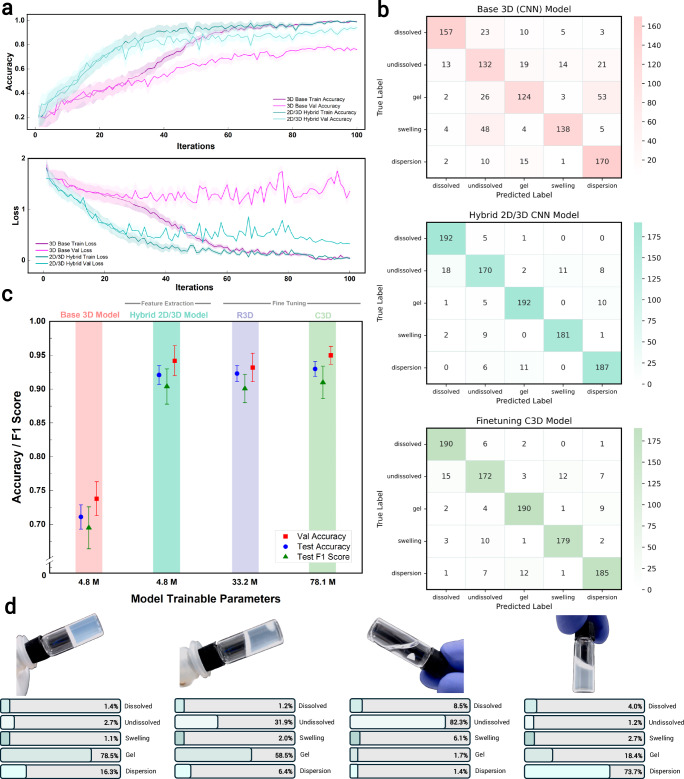
Comparative performance evaluation of 3D-CNN models and dynamic solvation state interpretation. **a** Training and validation accuracy and loss values during model training for a baseline 3D-CNN model (without finetuning) and the hybrid 2D-/3D-CNN model. **b** Confusion matrices of the baseline 3D model, hybrid 2D/3D-CNN model, and the fine-tuned C3D model. **c** Benchmarking results including validation accuracy, test accuracy, and F1 scores of the baseline 3D model, hybrid 2D-/3D-CNN model, and fine-tuned R3D and C3D model. **d** Identification of simultaneous phenomena in dynamic vial states using the hybrid 2D-/3D-model.

We first evaluated the feasibility of training a baseline 3D-CNN model from scratch using our domain-specific dataset. The model achieves average accuracies of 70–75%, highlighting the ability of the dataset to capture meaningful dynamic features within the proposed solvation classes and supporting the viability of domain-specific training using the 2D-/3D-CNN hybrid model architecture. Incorporating the 2D-CNN base model as a feature extractor in the hybrid architecture significantly improves performance, as shown in Fig. 3a, where the validation accuracy achieves significantly higher values with lower epochs, with similarly low validation loss. Moreover, as previously observed in the static inference models, fine-tuned 2D-CNN models outperform base static inference models across all metrics. When integrated into the hybrid architecture, these pretrained models further enhance performance, achieving classification results (validation accuracy of 0.938 at iteration 91 in Fig. 3a) that are comparable to, or exceed, those of pretrained 3D models such as R3D and C3D, as shown in Fig. 3c. While Fig. 3a shows a divergence between training and validation accuracy beyond iteration 91, indicating the onset of overfitting, the full 100-iteration training is intentionally shown to highlight the effectiveness of using features from the static 2D-CNN model to support the training of the dynamic inference model. In actual training, however, early stopping is employed to prevent overfitting and preserve generalisation performance. More importantly, the hybrid model achieves this while being approximately nine times more computationally efficient than R3D and sixteen times more efficient than C3D.

An analysis of the confusion matrices in Fig. 3b reveals the strengths and limitations of the baseline and the hybrid models. The baseline 3D-CNN model exhibits poor classification performance, similar to the base 2D model, particularly when distinguishing undissolved from swelling states and gel from dispersion states. In contrast, the hybrid model achieves significantly fewer misclassifications despite having the same number of trainable parameters (4.8 million) as the baseline 3D model. Even though the fine-tuned C3D model achieves a slightly higher F1 score of 0.92, it requires training 78.1 million parameters, making it significantly more computationally intensive than the hybrid model, which achieves a comparable F1 score of 0.91 with far fewer parameters. These results validate the use of hybrid models that leverage features extracted by 2D-CNNs to build 3D-CNN models for temporal and dynamic inference in the classification of polymer solvation states. This approach not only offers competitive performance but also maintains computational efficiency, making it highly suitable for resource-constrained environments.

Computer vision models inherently have the capability to provide probabilistic outputs through their softmax activation layers, which assign probabilities to each class during the inference step. However, in conventional classification configurations, only the class with the highest probability is output, limiting the model to a single-class prediction. For complex applications such as polymer solvation inference, leveraging these probabilistic outputs enables the identification of simultaneous phenomena and provides a deeper interpretation of overlapping solvation states. As shown in Fig. 3d, gelation can sometimes occur without fully dissolving the polymer; for example, a solvent may partially dissolve the polymer, increasing viscosity to the point of forming a gel. In such cases, instance-based classification might misinterpret the state as purely a polymer gel. By leveraging the dynamic inference module, the model can provide quantitative classification inferences, determining that the polymer predominantly exists as a gel (58.5%) while also recognising the presence of undissolved granules (31.9%) within the vial. This capability to account for overlapping solvation states enhances the interpretability of the results, offering a nuanced understanding of complex polymer–solvent interactions.

### Contextualisation of polymer solvation states

Polymer solvation states span a spectrum of physical conditions, making classification both inter-class and intra-class in nature. Inter-class classification involves distinguishing between distinct solvation states. In this study, we focused on five discrete classes: dissolved, undissolved, swelling, gelation, and dispersion. Intra-class classification, on the other hand, captures more subtle variations within these states. Polymers can exhibit diverse physical forms, appearing as powders, granules, or flakes with varying dispersion stability. In the case of copolymers, phase separation may occur at the molecular level, where one segment remains undissolved while another swells or forms colloidal domains, leading to complex intra-class distinctions in solvation behaviour. These variations reflect nuanced differences such as aggregation tendencies, surface interactions, or partial solvation, which are not explicitly delineated by the broader solvation categories. Polymer–solvent interactions add further visual complexity, as exemplified by lignin discolouring the solvent brown while remaining undissolved, or polyvinyl maleic anhydride forming a red or pink solution upon dissolution. These intricacies necessitate advanced interpretive tools capable of mimicking human expertise in contextualising both inter-class and intra-class polymer–solvent behaviours. Vision-language models hold significant potential in enabling this level of contextualisation, yet their adoption has been limited by the scarcity of domain-specific image–text datasets tailored to polymer solvation behaviours.

To move on beyond distinct classification states, we curated a dataset of 8011 image–text pairs representing a wide variety of polymer solvation states and conditions, providing context for both distinct and subtle variations. We used these captioned images to train a BLIP-2 model for domain adaptation to contextualising solvation states. However, finetuning the entire BLIP-2 model involves the training of more than 4 billion parameters, making it impractical for laboratory deployment in high-throughput screening workflows and rapid automation platforms. To address this, we utilised a parameter-efficient approach that combines the flattened concatenated vectors of feature maps generated by the 2D-CNN with feature embeddings from the BLIP-2 model. This method facilitates specific fine-tuning of the model, integrating the technical understanding of polymer solvation classes with the generic ability to contextualise and describe vial conditions. We benchmarked this approach using three configurations: models with two (~160M trainable parameters) or one (~80M trainable parameters) decoder layers unfrozen, and a configuration unfreezing only the fully connected layers (~52M trainable parameters).

**Fig. 4:**
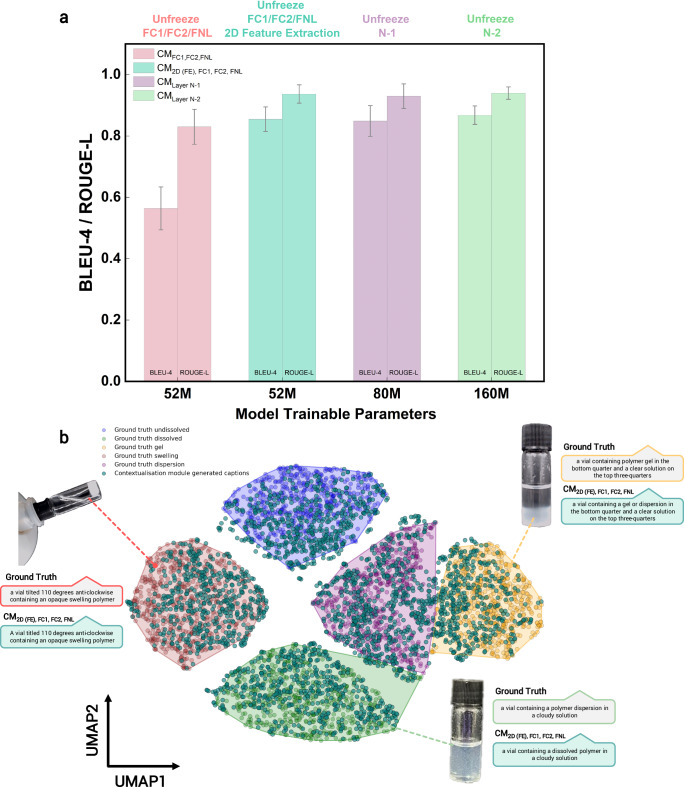
Evaluation of the contextualisation module using quantitative metrics and real-case scenarios. **a** BLEU-4 and ROUGE-L metrics for various contextualisation module (CM) configurations. CM_FC1, FC2, FNL_ refer to unfreezing the fully connected layers 1, 2, and the final normalisation layer of the language model. CM_2D (FE), FC1, FC2, FNL_ integrate 2D features extracted from the static inference module, concatenated with the unfrozen fully connected layers, before generating captions. CM_Layer *N*−1_ and CM_Layer N−2_ represent fine-tuned models with the last (*N*−1) and last two (*N*−2) language layers unfrozen, respectively. **b** UMAP visualisation of polymer solvation state captions, clustered by similarity in text embeddings. The contextualisation module’s performance is evaluated across various inference tasks, comparing ground truth captions with generated captions within different clusters.

Performance of the fine-tuned, repurposed vision-language model was evaluated using standard vision-language metrics, including BLEU-4^53^ and ROUGE-L^54^ scores. As shown in Fig. 4a, unfreezing the last two layers for fine-tuning in the captioning task achieves strong performance in describing solvation states, with BLEU-4 scores of 0.849 and 0.868 and ROUGE-L scores of 0.931 and 0.939. These notably high scores, exceeding typical benchmarks, are attributed to the use of a standardised set of captions during fine-tuning, such as ‘a vial containing undissolved polymer granules in a clear solution.’ The captions are designed to describe a wide range of outcomes, including distinct classification states, qualitative simultaneous phenomena, changes in the shades and colours of solvents, and the buoyancy or sedimentation of polymer entities. The consistency and specificity of these captions reduce linguistic variability and closely align the training, evaluation, and test datasets, enabling high overlap in word choice, sequence, and meaning. By evaluating the proposed training strategies, it becomes evident that the fine-tuned, repurposed vision-language model excels in generating accurate captions for the vials.

The parameter-efficient contextualisation strategy involving the incorporation of 2D features extracted from the static inference module further enhances the model’s ability to recognise visual patterns specifically related to polymer–solvent interactions. These patterns include textures and spatial arrangements that correlate with solvation states. In this approach, images processed by the static model produce a flattened, high-dimensional feature vector ([16, 86, 528]) containing domain-specific information. This vector is concatenated with feature embeddings from the vision-language encoder, which are optimised for caption generation and focus on localised image region details. The resulting embeddings align visual and textual representations, enabling the model to address domain-specific inquiries such as identifying polymer solvation states.

Beyond quantitative evaluation, a UMAP visualisation (Fig. 4b) of the text embeddings for ground-truth captions and generated captions reveal tight clustering, corresponding to the five polymer solvation classes depicted in this study. The close agreement between ground-truth captions and generated captions using the feature concatenation strategy highlights the model’s descriptive accuracy. However, as with all static modules, the contextualisation module, which operates on a frame-text inference basis, struggles to distinguish between gel and dispersion states, as reflected in the overlapping clusters for these categories. Analysis of edge cases further reveals challenges in applications when the vial contains a gel or a partial dispersion. Similarly, misinterpretation is observed between dissolved and dispersion states, where clarity of the vial leads to confusion in categorising and contextualising polymer solvation states. These issues are most prevalent for captions located between clusters or at the edge of their categorical clusters. Nonetheless, this tandem approach of supplying both feature maps and image–text embeddings to the decoder results in a parameter-efficient yet effective approach for providing context for the inference task. Its efficiency makes it highly suitable for deployment in laboratory settings and high-throughput workflows, where low computational overhead and rapid inference are essential.

## Discussion

Interpreting polymer solvation behaviours is a multifaceted challenge that requires context and often stretches beyond discrete categories. Polymer-solvent interactions can exist in a myriad of dynamic states, and this inherent complexity necessitates the capture of not only static solvation states but also temporal and morphological changes observed through behavioural manipulation. To address this, we have developed an extensive dataset comprising 13,663 images capturing static characteristics, 5053 videos of dynamic behaviours, and 8011 image–text captions annotating a wide range of solvation conditions. Using the dataset, a multi-model VA framework comprising three core components, namely static inference (2D-CNN), dynamic inference (hybrid 2D-/3D-CNN), and contextualisation (BLIP-2), was trained. These modules leverage parameter-efficient strategies in balancing computational efficiency with high classification accuracy, ensuring practical applicability in laboratory environments.

The static inference model was trained using a 2D-CNN approach, forming the foundation of the VA framework. While the 2D-CNN baseline model does not outperform pretrained fine-tuned models like DenseNet50 or ResNet50 in traditional classification tasks, its significance lies in its role as a feature extractor. The interpretable feature maps generated by the 2D-CNN effectively capture morphological and contextual details of solvation states, forming a critical foundation for downstream modules. These feature maps allow the 2D-CNN to act as a bridge that translates static solvation behaviours into embedded insights that support both dynamic inference and contextualisation tasks. The ability to extract useful information enables the development of bespoke methods tailored to tackle the challenges of the polymer solvation domain.

Understanding polymer solvation behaviours requires capturing both static and dynamic characteristics, as many phenomena evolve over time and cannot be fully discerned from static images alone. For instance, gels exhibit viscosity-driven flow patterns, while dispersions show settling behaviour over time. To address this, the hybrid 2D-/3D-CNN architecture leverages static features extracted by the 2D-CNN, significantly reducing the parameter requirements of traditional 3D-CNNs while achieving competitive or superior performance. Compared to pretrained R3D or C3D models, the 2D-/3D-CNN hybrid achieves nearly tenfold parameter efficiency without compromising accuracy. This model excels in classifying dynamic solvation states, capturing simultaneous phenomena with remarkable granularity. These findings highlight the potential of hybrid architectures to deliver robust and efficient solutions for complex, temporally dependent classification tasks.

The BLIP-2 contextualisation module enhances solvation state evaluation by generating descriptive captions that closely align with human assessments. Our approach integrates 2D-CNN-extracted features with BLIP-2 encoder embeddings, improving the model’s ability to generate nuanced and domain-specific captions while significantly reducing computational cost compared to more resource-heavy finetuning methods. Specifically, we unfreeze either one (~80M parameters) or two (~160M parameters) decoder layers, enabling the model to adapt to polymer–solvent-specific tasks while remaining computationally efficient compared to full model fine-tuning. This approach allows the contextualisation module to interpret complex interactions, such as colour changes in the solvent or morphological shifts in the polymer, without increasing computational load or compromising accuracy. The results demonstrate that this feature concatenation strategy balances interpretability and computational efficiency, making it suitable for laboratory deployment.

Further refinements could enhance the capability of the vision assistant, particularly in leveraging temporal dependencies more effectively. Since BLIP-2 primarily operates on image-caption pairs, it faces challenges similar to the static inference model in distinguishing between dispersive and gel conditions in certain cases. Expanding the number of discrete solvation classes could also improve granularity, though this would require a more extensive dataset and additional model refinement to ensure robust classification. Advanced video-based vision-language models or transformer-based approaches could provide deeper contextualisation of dynamic solvation behaviours, yet their reliance on large-scale video annotations presents challenges in data labelling and computational overhead. Alternative CNN architectures could offer improved temporal feature extraction, which is crucial for capturing subtle phase transitions in polymer–solvent interactions, with careful evaluation of the trade-offs between performance and computational requirements. Exploring self-supervised and contrastive learning techniques may further mitigate the dependence on large, labelled datasets, improving generalisability while efficiently managing computational demands. These potential advancements, while beyond the current scope, highlight promising directions for future research and reinforce the need for diverse and well-annotated datasets in polymer sciences to support the development of scalable AI-driven solvation models.

The methodologies and datasets presented here establish a framework for advancing polymer science through automation and high-throughput analysis. By leveraging image analysis, the VA overcomes the limitations of subjective human observation, enabling accurate, reproducible, and automated workflows for polymer–solvent interaction evaluation. Beyond solvation states, the VA’s modular design offers adaptability to other classification challenges, such as colloidal stability, phase separation, or crystallisation behaviours. The emphasis on parameter-efficient architectures ensures quick adaptation across laboratories with varying computational capacities. Specific applications could extend these datasets and models to incorporate additional classification domains such as colloidal systems and phase separation. By addressing the bottlenecks in polymer–solvent interaction evaluation, this study paves the way for more nuanced, scalable, and efficient scientific enquiry, complementing human expertise with machine intelligence.

## Methods

### Workflow for generating polymer solvation dataset

To train the VA, we undertook a large-scale data generation campaign, testing 200 unique homo- and co-polymers against 36 solvents at four distinct temperatures (4, 25, 50, and 75 °C). This comprehensive approach aimed to capture diverse polymer–solvent interactions, including behaviours, such as dissolution, swelling, and dispersion, under realistic laboratory conditions.

To ensure consistency and efficiency, we developed a semi-automated workflow that combined robotic and manual methods. Polymers were dispensed into 1.5 mL clear glass vials using a ChemSpeed (SWILE) robotic system equipped with glass dispensing tools, which enabled precise and rapid dosing of powders. For polymers in flake or granule forms, manual dosing was employed due to compatibility limitations with the robotic system. Solvents were dispensed into 25 mL clear glass vials using an OpenTrons OT-2 liquid handling robot, ensuring accurate and reproducible measurements. To accommodate the solvents and polymer-containing vials, custom racks were 3D printed from acrylonitrile butadiene styrene, chosen for their chemical resistance to common solvents.

Each polymer–solvent combination was prepared at a concentration of 2.5 wt./vol%, allowing for clear visual identification of solvation behaviours. Once the solvents were added, the vials were placed in a Corning LSE 71 L shaking incubator and agitated at 300 RPM for 24 h under precisely controlled temperatures (±0.5°C). Following agitation, the vials were removed and photographed under ambient laboratory lighting. This ensured that the dataset reflected typical laboratory conditions, removing the need for specialised lighting setups.

To maximise diversity within the dataset, we included a wide range of polymer species, including polystyrene, polymethyl methacrylate, polyacrylic acids, polyvinyl chloride, polyvinyl acetate, polyvinylpyrrolidone, and polyvinyl alcohol. The polymers were represented in various physical forms, such as powders, granules, and flakes. Additionally, we tested these polymers against a selection of industrial solvents, such as alcohols, ketones, and hydrocarbons, to capture a broad range of solvation behaviours, including dissolution, swelling, gelation, and dispersion.

Images were acquired using a range of devices, including a Logitech C920 webcam, iPhone 13, and iPhone 14 Pro Max, saved in .jpg format with resolutions of 3024 × 4032 pixels. To ensure consistency, all images were taken either within a portable photography chamber or a benchtop platform equipped with a black background to enhance contrast and minimise background distractions. Photos were taken using varying focal lengths and angles. These variations were deliberately introduced to account for potential distortions or differences in perspective that may arise in practice. Images were further enhanced by adjusting contrast and brightness settings to compensate for variability in lighting conditions across different laboratory environments. To ensure uniform illumination and reduce harsh shadows, diffused lighting was employed. This setup aimed to achieve an even distribution of light across the samples while preserving subtle visual details crucial for accurate classification.

Videos were captured exclusively using the Logitech C920 webcam at a resolution of 1920 × 1080, sampled at 30 fps for consistency. To capture dynamic solvation behaviours, such as gel formation, dispersion, and swelling, two approaches were employed. Manual handling involved researchers agitating, tilting, and shaking the vials to simulate standard laboratory practices, introducing realistic variability. In contrast, a robotic arm was programmed to hold and tilt the vials along their axis, providing standardised and reproducible movements. Diffused light from below the vials in ambient light conditions replicated real laboratory environments, ensuring optimal visibility of solvation behaviours. This combination of manual and robotic handling resulted in a diverse and comprehensive dataset, well-suited for integration with self-driving laboratory systems.

This workflow generated a comprehensive dataset of polymer–solvent interactions, capturing both static (image-based) and dynamic (video-based) behaviours. The dataset formed the foundation for training the VA’s static inference module (2D-CNN) and dynamic inference module (3D-CNN), enabling the accurate classification and interpretation of polymer–solvent behaviours.

### Static inference module

The static inference module was designed to classify polymer–solvent interactions using a dataset of high-resolution images captured under controlled conditions. Among the dataset of 13,663 images, 4875 were identified and manually classified into five distinct categories representing common polymer–solvent interactions: dissolved, undissolved, swelling, gel, and dispersion. To achieve balanced representation across these categories, a selective sampling approach was employed. Within each class, a wide range of representative images was selected for model training to ensure diversity in solvation behaviours and improve generalisability. This strategy mitigated potential model bias and ensured optimal performance during training.

The dissolved class consisted of 925 images of clear liquids with no visible solid particles or phase separation. The undissolved class included 1070 images showing solid polymer powders or granules that maintained their original shape, size, and optical properties. The swelling class contained 960 images depicting solid polymers that had visibly changed in shape, size, or optical properties due to partial solvation. The gel class featured 861 images of semi-solid, jelly-like materials occupying parts of the vial or forming a distinct layer at the bottom. Finally, the dispersion class comprised 1059 images of cloudy liquids where the polymer was finely dispersed in the solvent, with undissolved powders suspended and sometimes settling over time. These categories were chosen based on their prevalence and importance in polymer–solvent systems.

A base 2D-CNN model was developed to classify the dataset. The input images were resized to 224 × 224 pixels with three colour channels. The architecture consisted of three convolutional layers. The first layer included 32 filters with a (3, 3) kernel size and ReLU activation, followed by a MaxPooling layer with a (2, 2) pool size. The second layer had 64 filters with the same kernel size and activation function, followed by another MaxPooling layer. The third convolutional layer incorporated 128 filters and a MaxPooling layer. After feature extraction, the output was flattened and passed through a fully connected layer with 32 units, followed by a Dropout layer with a rate of 0.05 to reduce overfitting. The final output layer used a softmax activation function to classify the input into the five defined categories. The model was compiled using the Adam optimiser with a learning rate of 0.001, categorical cross-entropy as the loss function, and accuracy as the performance metric.

In addition to the base 2D-CNN model, several pre-trained architectures were fine-tuned for comparison. These included VGG16, InceptionV3, ResNet50, and DenseNet121, each initialised with ImageNet weights. The convolutional layers of these models were frozen to retain the robust feature representations they had learned from large-scale image datasets. Fine-tuning was restricted to the final layers, enabling the models to adapt to the polymer–solvent classification task. The pre-trained models varied in architecture: VGG16 employed five convolutional blocks, InceptionV3 utilised inception modules for multi-scale feature extraction, ResNet50 leveraged residual connections to mitigate vanishing gradients, and DenseNet121 incorporated densely connected layers to enhance gradient flow. Following feature extraction, the outputs were flattened and passed through a fully connected layer with L2 regularisation and a mild Dropout rate of 0.05, concluding with a softmax-activated dense layer for classification. All static inference models using 2D-CNN architectures were trained for up to 50 epochs with an early stopping patience of 5 epochs to prevent overfitting.

### Dynamic inference module

The dynamic inference module addresses the limitations of static image-based analysis in determining polymer–solvent solvation states by taking in video inputs for classification. Static images often fail to capture transient phenomena such as swelling, which involves the gradual enlargement of polymers as solvent molecules infiltrate their chain networks. Similarly, behaviours such as phase separation or gel formation may only become evident when the vial is tilted or agitated, revealing underlying dynamics that remain concealed in static states. For example, complex interactions can involve coexisting dispersion and gelation, where static inference may identify only the dominant state without capturing the complete behaviour. Manual handling practices by researchers, such as shaking or tilting vials, are integral for identifying such nuances. Thus, a dynamic inference approach that incorporates temporal and mechanically induced movement behaviours enables a more accurate replication of manual observational techniques, facilitating improved solvation state identification.

To achieve this, a 3D-CNN was developed for analysing video data. Unlike static models, the 3D-CNN processes sequences of video frames, allowing it to detect temporal changes and track dynamic behaviours. Each frame is treated as an individual input, and the model learns to identify variations in feature maps over time. A dataset comprising 5053 videos collated amongst the captured video data was evenly distributed across the five proposed polymer solvation classes to train the 3D-CNN. To ensure uniformity, the vials were centred in the video frames during recording. Further, the dataset was enriched with two distinct handling methods: robotic and manual. A robotic arm rotated vials along their central axis, capturing all observable angles, while human operators introduced natural variability by shaking and tilting the vials. These manual interactions included slight deviations along the *x*-axis due to hand movement, offering a diverse range of inputs. This combination of robotic and manual manipulation enabled the model to generalise better to real-world scenarios. Horizontal flips were applied to the video inputs to enhance robustness against geometric variations. Videos were resized to 224 × 224 pixels for training. A frame sampling rate of 30 frames per second was used to ensure detailed temporal dynamics, allowing the model to distinguish between behaviours like slow viscous gel flow and faster dispersion dynamics.

The architecture of the base 3D-CNN consisted of five 3D convolutional layers. The first layer applied 32 filters with a kernel size of (3, 3, 3) and ReLU activation, followed by a MaxPooling3D layer with a pool size of (1, 2, 2) to focus on spatial features. Subsequent layers increased the filter count to 64, 128, 256, and 512, with MaxPooling3D layers configured to incorporate spatial and temporal pooling in later blocks. Dropout rates ranging from 0.1 to 0.2 were included after each layer to prevent overfitting. The feature maps were flattened through a GlobalAveragePooling3D layer, followed by a dense layer with 256 units and ReLU activation. A final dense layer with softmax activation generated the probabilistic outputs for the five solvation classes. The model was compiled using the Adam optimiser with a learning rate of 0.001 and categorical cross-entropy loss. The dynamic inference models using 3D-CNN or 2D/3D-CNN hybrid architectures were trained for up to 100 epochs with early stopping applied at patience of 10 epochs to avoid overfitting.

To benchmark performance, a hybrid 2D-/3D-CNN model was also developed. The 2D-CNN from the static inference module was incorporated using a time-distributed layer to apply spatial feature extraction on each video frame, followed by an LSTM layer with 512 units for temporal modelling. This hybrid architecture allowed the model to leverage the strengths of both spatial and temporal feature learning. Pre-trained models, such as R3D and C3D, were fine-tuned to further compare performance against the hybrid 2D-/3D-CNN model. These models were initialised with pre-trained weights and fine-tuned on the video dataset, with their convolutional layers frozen to retain learned representations while adapting the final layers to the polymer–solvent classification task.

The dynamic inference approach demonstrated superior performance in accurately identifying solvation behaviours by capturing temporal and agitation-dependent characteristics. By combining 3D-CNN architectures, hybrid models, and robust benchmarking, this module provides a comprehensive solution for analysing complex solvation states that are not discernible through static inference alone.

### Contextualisation module

The contextualisation module employs a vision-language model to enhance interpretability by generating descriptive captions for vial images, specifically tailored to polymer–solvent interactions. Domain adaptation was performed on the BLIP-2 model using a custom dataset of 8011 image-caption pairs. These captions describe solvation behaviours observed in vials, such as dissolved, undissolved, gel, swelling, or dispersion states. BLIP-2, pre-trained on large-scale multi-modal datasets like Common Object in Context (COCO) and Visual Genome, was fine-tuned to specialise in polymer–solvent interactions.

To adapt the model, several configurations were benchmarked to balance computational efficiency with the ability to capture domain-specific nuances. In the first configuration, the last two layers of the language model were unfrozen, resulting in 160 million trainable parameters, enabling the model to adapt more extensively for generating detailed captions. The second configuration reduced computational demands by unfreezing only the last layer, resulting in 80 million trainable parameters. A third configuration focused on unfreezing the fully connected layers (FC1 and FC2) and the final normalisation layer, reducing the trainable parameters to 52 million. Finally, a hybrid approach was explored in which feature maps extracted from a separately trained 2D CNN were integrated into the BLIPv2 model while maintaining the unfreeze configuration of the FC1, FC2, and final normalisation layer. This integration enriched the model’s visual representations with domain-specific features, improving caption quality without significantly increasing computational requirements.

Training for all configurations was implemented in PyTorch on Google Colab Pro+, using either an NVIDIA L4 or NVIDIA A100 GPU. This setup balanced the necessary computational power with cost-effectiveness, enabling efficient model development without requiring dedicated HPC infrastructure. A batch size of sixteen was used for the training set and eight for the validation and test set. The dataset was split into 70% for training, 15% for validation, and 15% for testing, ensuring robust evaluation. Preprocessing steps included resizing images to 224 × 224 pixels, normalising pixel values, and tokenising captions into sequences of tokens for model input. Data augmentation techniques, such as brightness and contrast adjustments, simulated varied lighting conditions to improve robustness. The AdamW optimiser, combined with weight decay regularisation and dropout layers, was used to mitigate overfitting. A learning rate scheduler, initialised at 0.00001, applied a decay factor of 0.1 if validation loss did not improve for three consecutive epochs. An early stopping mechanism terminated training after five epochs without improvement.

Performance evaluation utilised bilingual evaluation understudy (BLEU-4) and n-gram recall (ROUGE-L) metrics to assess the semantic alignment and coherence of the generated captions against ground-truth annotations (Fig. 1b). The BLEU-4 score is a metric for evaluating the quality of text by comparing it to one or more reference texts and can be computed with Eq. (1).

1 $${{\rm {BLEU}}}-4={{\rm {BP}}}\cdot \exp \mathop{\sum }\limits_{n=1}^{4}{w}_{n}\cdot \log {p}_{n}$$

${{\rm {BP}}}$ refers to the brevity penalty defined as: $${{\rm {BP}}}=\left\{\begin{array}{c}1\\ {{\rm {e}}}^{(1-\frac{r}{c})}\end{array}\right.\left\{\begin{array}{c}{{\rm {if}}\; c} > r\\ {{\rm {if}}\; c} < r\end{array}\right.$$ where $c$ refers to the total length of candidate captions (generated output) and $r$ refers to the total length of reference captions (ground truth). ${w}_{n}$ are the weights assigned to the n-gram precision and the ${p}_{n}$ is the n-gram precision, calculated with ${p}_{n}=\frac{{{\rm {Count}}\; {\rm {of}}\; {\rm {overlapping}}\; n}-{{\rm {grams}}}}{{{\rm {Total}}\; {\rm {number}}\; {\rm {of}}\; n}-{{\rm {grams}}\; {\rm {in}}\; {\rm {candidate}}}}$. The ROUGE-L equation evaluates the overlap of the longest common subsequence (LCS) between the generated caption and the reference caption. ROUGE-L computes a type of F1-score using precision and recall derived from the LCS and is expressed mathematically in Eq. (2).

2 $${{\rm {ROUGE}}}-L=F-{{\rm {score}}}=\frac{(1+{\beta }^{2})\cdot {{{\rm {Precision}}}}_{{{\rm {LCS}}}}\cdot {{{\rm {Recall}}}}_{{{\rm {LCS}}}}}{{\beta }^{2}\cdot {{{\rm {Precision}}}}_{{{\rm {LCS}}}}+{{{\rm {Recall}}}}_{{{\rm {LCS}}}}}$$

$\beta$ refers to a weight that balances the importance of recall and precision, for the purposes of this evaluation, we have set $\beta$ = 1, giving equal importance to both recall and precision. Precision is a measure of how much the caption text overlap with the reference caption and the recall measures how much of the reference caption is captured in the generated caption sequence.

### Types of polymers solvation behaviours

It is important to recognise that polymers can exist in various physical forms, such as granules, resin, powder, flakes, and beads, depending on their synthesis pathways or product requirements. The same polymer, when presented in different physical forms, may exhibit distinct solvation dynamics. However, while it may not always be essential to identify the specific physical form, it is crucial to establish consistency in observing and interpreting the visual cues associated with each type of solvation behaviour. For the purposes for this study, polymer solvation behaviours are segregated into five-distinct classes: dissolved, undissolved, swelling, gel, and dispersion.

A dissolved behaviour refers to a process where the polymer is completely dispersed at the molecular level within the solvent, resulting in a homogeneous solution. In this state, the polymer chains are surrounded by solvent molecules, leading to no visible solid residue or phase separation. The viscosity of the solution remains relatively unchanged, meaning the solution continues to behave like the original solvent, maintaining its low resistance to flow. In other words, the system appears clear, runny, and no polymer particles are observable, indicating complete dissolution.

Undissolved cases refer to systems where the polymer remains insoluble in the solvent, maintaining its original structure without significant interaction. In these situations, the polymer does not dissolve or swell and remains intact within the solution. The polymer granules, beads, or powders can settle at the bottom of the vial or remain suspended as visible solid particles that do not change size or exhibit surface modifications. Additionally, if the polymer’s density is lower than that of the solvent, the granules, powders, or beads may float on the surface of the vial. These systems often display clear phase separation, where the solvent remains transparent, and the polymer does not contribute to cloudiness or noticeable changes in viscosity. Despite exposure to the solvent, the polymer does not interact chemically or physically in a manner that would lead to dissolution or swelling.

In this study, swelling describes the partial interaction between the polymer and the solvent, leading to significant physical changes in the polymer. Swelling is evidenced by polymer granules becoming sticky and adhering to the walls of the vial or by an increase in the granules’ size as they absorb the solvent. Additional signs of swelling include visible cracks in the polymer structure after prolonged solvent exposure, or polymeric beads coalescing into chains. Swelling can also result in heterogeneous layers forming at the bottom of the vial or beads and powders sticking throughout the vial’s surface, resisting agitation. Often, swollen polymers are dispersed within the solvent, creating a colloidal suspension where the polymer particles remain partially swollen and suspended in the solvent.

Gel formation represents a smaller proportion of the observed solvation behaviours. Gelation is characterised by a noticeable increase in viscosity as the solvent dissolves the polymer, resulting in a thickened solution with high resistance to flow. This thicker appearance is further accompanied by the formation of gels and blobs, which visibly phase-separate from the solvent as the polymer breaks down into a liquid form. Shaking or agitation reveals gelation tendencies, with blobs detaching at higher intensities, while tilting shows the gel layer flowing more slowly than the phase-separated solvent. Additionally, the gel is defined in this context as a solid-like substance, like a cloth, which retains a flexible but structured form, capable of holding liquid within its network.

For the purposes of this study, the dispersion class, includes colloidal systems, emulsions, and suspensions, and refer to systems where solid particles, such as polymers, are finely distributed within a liquid solvent. Colloidal systems involve particles typically in the nanometer to micrometer range that remain suspended without settling, while emulsions involve immiscible phases where one is dispersed as droplets in the other. Suspensions consist of larger solid particles dispersed in a liquid and may settle over time. Dispersion often occurs alongside other phenomena, such as a powder making the solution appear cloudy while remaining undissolved, or polymeric beads swelling in the solvent, creating a cloudy solution with suspended beads. These systems share visual characteristics such as turbidity and phase separation, which are key for identifying solvation behaviours.

## Supplementary information

Supplementary Information

## Data Availability

All data are uploaded onto an online repository (https://zenodo.org/records/14319147). The repository contains 13,663 static images representing a wide range of polymer solvation behaviours. It includes 5053 video files reflecting dynamic behaviours of polymer-solvation behaviours mechanistically by human or a robot arm. Over 8011 image–text captions for training vision-language models. The static images and image–text captions are not uniquely overlapping, image–text captions contain more contextualised heavy variations and some extracted frames from the video files.

## Code availability

The executable python code and documentation to achieve the different modalities of the VA to infer polymer-solvation behaviours are available via GitHub at (https://github.com/zjlucam/VisionAssistant). We provide a full list of software dependencies in the corresponding repositories. The code is provided without restrictions under the MIT license. All models were trained using compute resources provided by Google Co-Lab, either Nvidia A100 GPUs with 40GB memory or L4 GPU with 24 GB was used to train and fine-tune all 2D-CNN, hybrid 2D/3D-CNN, 3D-CNN, and BLIP-2 models.
