# Supplementary material for: Parameter efficient multi-model vision assistant for polymer solvation behaviour inference
Source: NPJ Comput Mater. 2025 May 31;11(1):161. doi: 10.1038/s41524-025-01658-7 (PMC12126306; doi:10.1038/s41524-025-01658-7)
Supplement: Supplementary file 1 — Supplementary Information [file 41524_2025_1658_MOESM1_ESM.pdf]

# Supplementary Information

# Parameter Efficient Multi-Model Vision Assistant for Polymer Solvation Behaviour Inference

Zheng Jie Liew,[1,2] Ziad Elkhaiary,[1] and Alexei A. Lapkin[1,2]

[1] Department of Chemical Engineering and Biotechnology, University of Cambridge, Cambridge CB3 0AS, U.K.

[2] Innovation Centre in Digital Molecular Technologies, Yusuf Hamied Department of Chemistry, University of Cambridge, Cambridge CB2 1EW, U.K.

**Table S1.** Detailed runtime comparisons based on data loading strategies for training the vision assistant using Google Colaboratory CPU and GPU services. The dataset used for these measurements corresponds to the modules described in the main text. *Not feasible* indicates that 3D video data and BLIP models are typically too large to pre-load entirely into memory, particularly when working with multi-video datasets. However, in limited cases involving small datasets, pre-loading may be possible on A100 GPUs with 80 GB of VRAM. Batch size, dataset size, and number of training epochs follow the specifications outlined in the main text.

| Inference Type | Static Inference (2D-CNN) | Dynamic Inference (3D-CNN) | Contextualisation (BLIPv2) |
|---|---|---|---|
| **CPU** | | | |
| Runtime (single loading) | 30 mins | 4 hours | 2 hours |
| Runtime (parallel loading) | 20 mins | *Not feasible* | *Not feasible* |
| Runtime (pre-loading into memory) | *Not feasible* | *Not feasible* | *Not feasible* |
| **L4 GPU** | | | |
| Runtime (single loading) | 3 – 5 mins | 20 – 30 mins | 10 – 15 mins |
| Runtime (parallel loading) | 2 – 3 mins | 15 – 20 mins | 8 – 10 mins |
| Runtime (pre-loading into memory) | 1 – 2 mins | *Not feasible* | *Not feasible* |
| **A100 GPU** | | | |
| Runtime (single loading) | 1 – 2 mins | 8 – 10 mins | 4 – 6 mins |
| Runtime (parallel loading) | < 1 min | 5 – 8 mins | 3 – 5 mins |
| Runtime (pre-loading into memory) | < 1 min | 3 – 5 mins | *Not feasible* |

**Table S2.** Comparison of model complexity and training runtimes for the Hybrid 2D–3D CNN model, R3D, and C3D across CPU and GPU hardware configurations. Runtime refers to total training time using single data loading, without parallelisation or memory pre-loading. All models were trained under consistent conditions, with identical batch size, dataset size, and number of epochs as specified in the main text. The hybrid model demonstrates substantial computational efficiency while maintaining competitive performance.

| **Model Type** | **# Trainable Parameters** | **CPU Runtime** | **A100 Runtime** | **Notes** |
|---|---|---|---|---|
| Hybrid 2D-3D CNN | 4.8 M | 5 hours | 8 – 10 mins | 2D feature extractor + shallow 3D |
| R3D-Based | 63 M | 6 – 7 hours | 20 – 30 mins | Deep 3D model |
| C3D-Based | 78 M | 8 – 9 hours | 25 – 35 mins | Legacy model, high compute cost |
